# Clarity Amidst the Smoke: Moyamoya Disease, a Diagnosis Not to Be Missed

**DOI:** 10.7759/cureus.78810

**Published:** 2025-02-10

**Authors:** Sharifa Alnaqeeb, Diana Kheder, Noor A Abotaiban

**Affiliations:** 1 Internal Medicine, Farwaniya Hospital, Kuwait City, KWT; 2 Neurology, Kasralainy Hospital, Cairo University, Cairo, EGY

**Keywords:** case report, cerebral angiography, ischemic and hemorrhagic stroke, moyamoya disease (mmd), surgical revascularization, transient ischemic attack (tia)

## Abstract

Moyamoya disease (MMD) is a rare, progressive cerebrovascular disorder characterized by the bilateral stenosis of the internal carotid artery (ICA) and its proximal vessels, leading to the formation of compensatory smoke-like collateral vessels. This report describes the case of a 33-year-old Philippine woman who presented with acute right-sided hemiparesis and had a history of recurrent transient ischemic attacks (TIAs) with similar symptoms. Neuroimaging revealed intracranial hemorrhage and characteristic bilateral occlusion of the internal carotid artery (ICA), middle cerebral artery (MCA), and anterior cerebral artery (ACA) with collateral vessel formation, indicative of moyamoya disease. The patient was referred for surgical revascularization.

## Introduction

Moyamoya disease (MMD) is a rare cerebrovascular disorder characterized by bilateral progressive stenosis of the internal carotid artery (ICA), middle cerebral artery (MCA), and anterior cerebral artery (ACA) [1]. This leads to neovascularization and the formation of smoke-like collateral vessels to compensate for the occlusion [2]. It shows an epidemiological distribution that favors East Asian populations, having a higher incidence in Japan, Korea, and China, and is almost twice as common in females [2]. Patients usually present with symptoms of an ischemic insult or cerebral hemorrhage [3]. Diagnosis is established through cerebral angiography, while CT and MRI are used for further evaluation. Recent criteria have also been updated to include unilateral presentations [1,3]. Treatment mainly involves surgical revascularization, with antiplatelet therapy used in some cases [4].

## Case presentation

**Figure 1:**
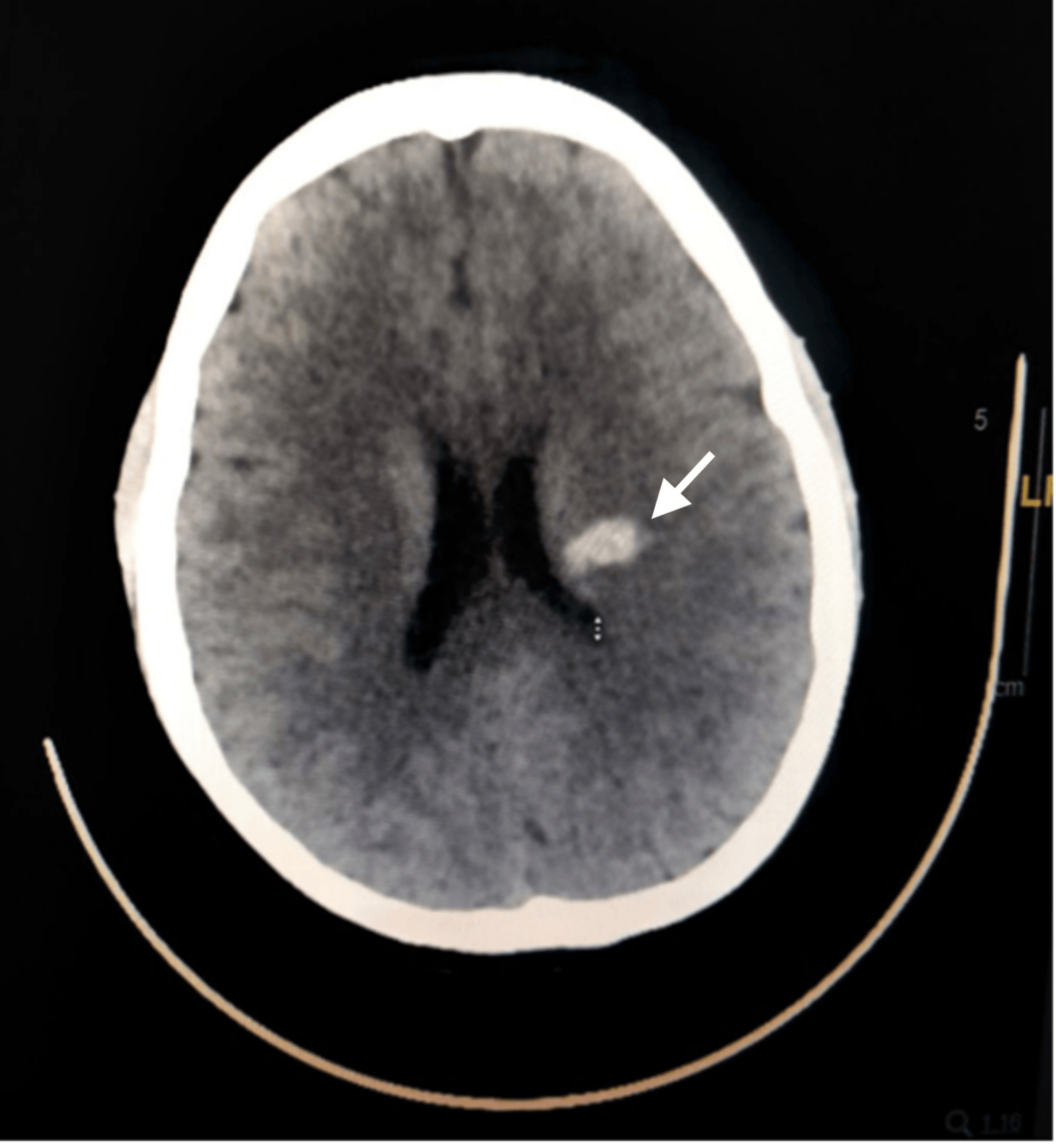
Axial CT brain depicting the presence of a left sided high parietal hematoma

A 33-year-old Philippine woman with a medical history of hypertension presented to casualty with acute onset right-sided facial weakness and hemiparesis. Initially, she was admitted as a case of suspected stroke within the window. Neuroimaging concluded the presence of a left-sided basal ganglia hematoma (Figure 1), and she was subsequently admitted to the ICU for conservative management as a case of hypertensive emergency complicated by intracranial hemorrhage. Upon examination, she was found to have right-sided pyramidal weakness along with dysarthria. 

Further examination of her medical history revealed that she had suffered from multiple episodes of transient ischemic attacks (TIAs), manifesting in the form of right-sided weakness primarily affecting the upper limbs more extensively than the lower ones. She denied any history of previous cerebrovascular accidents (CVAs), illicit drug use, joint pain, skin rash, or thromboembolic events. She had three uneventful full-term pregnancies and denied any family history of similar events.

**Figure 2:**
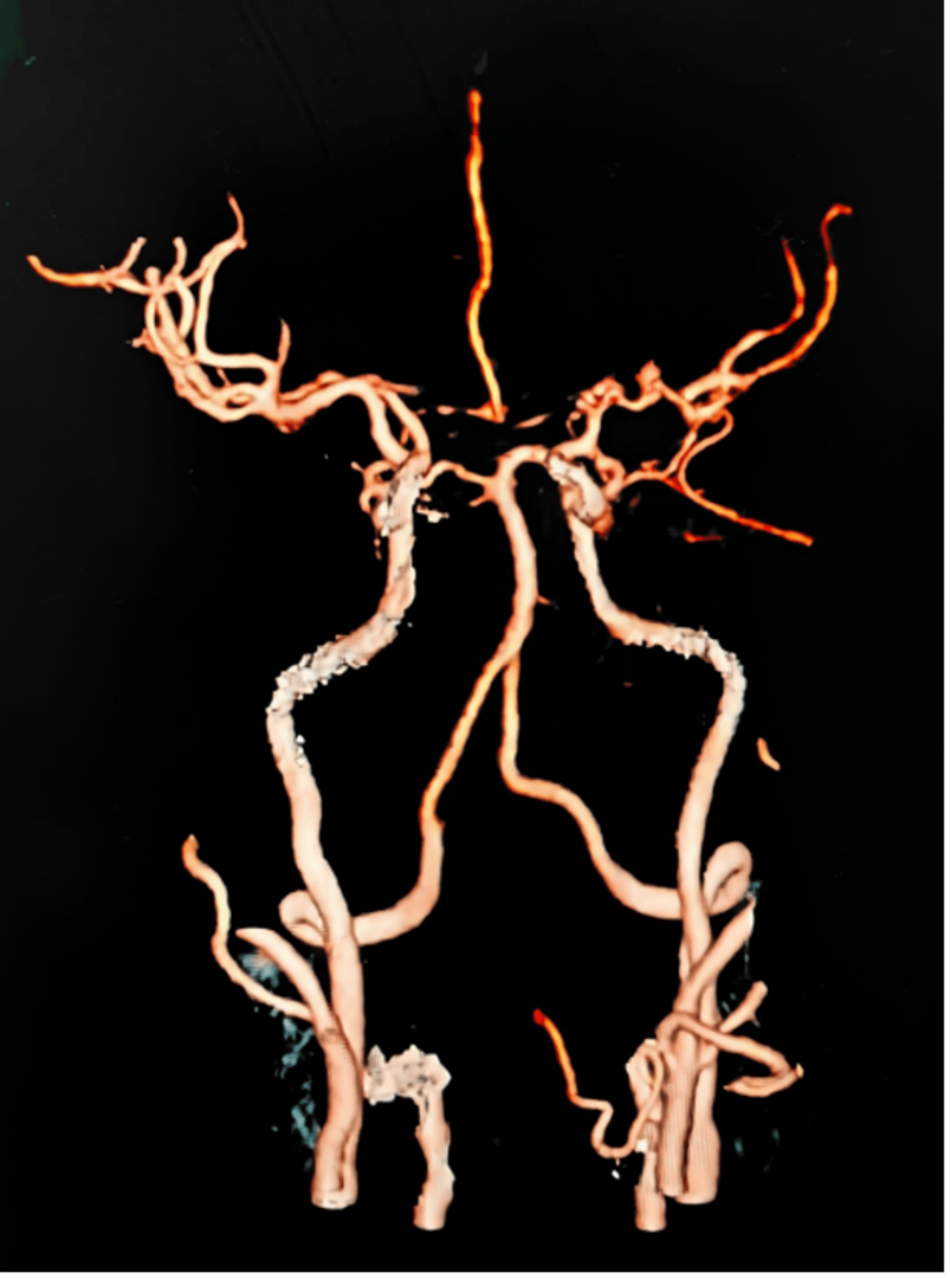
MRA showing bilateral stenosis in distal ICAs and proximal MCAs. ICAs: internal carotid arteries; MCAs: middle cerebral arteries.

**Figure 3:**
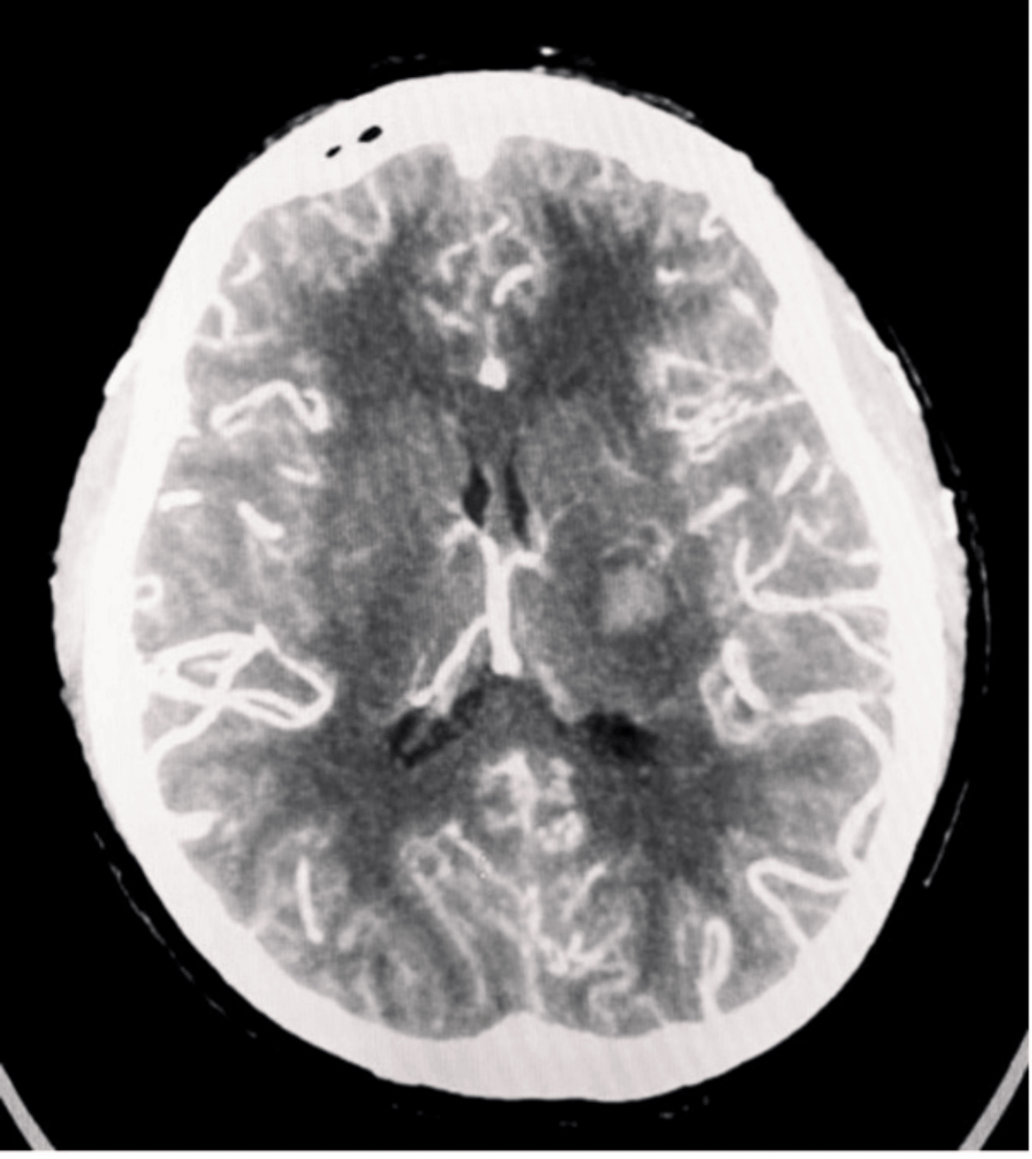
Axial CT angiography showing extensive collateral circulation with picture of 'puff of smoke'.

As part of the stroke workup, the patient underwent CT angiography, which showed bilateral occlusion of the ICAs, ACAs, and MCAs (Figure 2) and the presence of collateral circulation in addition to the basal ganglion acute hematoma (Figure 3). The rest of her stroke workup, including an autoimmune screen, was negative. She was referred to the neurosurgery department for surgical re-vascularization but was ultimately managed conservatively. 

## Discussion

Due to chronic cerebrovascular occlusion of the ICAs, MCAs, and ACAs, the pathophysiology of MMD involves the formation of a collateral vasculature network [2]. Moyamoya disease was named for the resemblance of its collateral vessels to a puff of smoke [5]. The annual incidence in Japan is estimated to be around 0.94 per 100,000 people, with a prevalence of 10.5 per 100,000 [6]. MMD exhibits a bimodal age distribution, with one peak around 5-10 years old and another around 30-40 years old [2]. This distribution reflects different clinical presentations and disease progression patterns. 

Although the etiology of MMD is not clear, genetic factors are believed to play a significant role, particularly in East Asian populations, where around 12% of MMD patients have a positive family history [7]. A genome-wide associated study identified the p.R4859K mutation on the RNF213 locus as a major risk factor for this disease [8]. It has been hypothesized that underlying chronic inflammation may promote the progression of MMD, especially in the case of Moyamoya syndrome [9]. Moyamoya syndrome is diagnosed when MMD occurs in association with other conditions, such as meningitis, autoimmune disease, brain tumors, Down’s syndrome, neurofibromatosis type 1, and head irradiation [10].

Patients with MMD typically present with ischemic or hemorrhagic strokes, transient ischemic attacks, seizures, or cognitive dysfunction [2]. Children typically present with progressive ischemic symptoms, while about half of the adult presentations are hemorrhagic [11]. The gold standard for diagnosis is catheter cerebral angiography, although less invasive techniques are available, such as CT angiography, MRI, and magnetic resonance angiography (MRA) [4]. These studies provide images of the characteristic stenosis and collateral vessel formation. 

In the acute phase, standard guidelienes for the management of stroke are followed. The cornerstone of of treatment for MMD is surgical revascularization, which can be direct, indirect, or combined bypass procedures [1]. Revascularization helps restore cerebral perfusion and prevent further strokes and has been shown to improve long-term outcomes [12-14]. Medical management focuses on secondary prevention of stroke and symptom management through the use of anti-platelets like aspirin or clopidogrel [1]. However, studies on the benefit of anti-platelets have shown mixed results [15-22].

The prognosis of MMD depends on the severity of vascular occlusion and the implementation of appropriate treatments. Conservatively treated patients with MMD are more likely to expereince subsequent strokes, TIAs, and progression of the disease [23]. Those presenting with hemorrhagic strokes have lower 10-year survival rates [24].

## Conclusions

Although it is a rare condition, this case highlights the importance of recognizing MMD in young patients presenting with stroke-like symptoms, particularly in populations with a higher prevelance of the disease. Early diagnosis and appropriate surgical intervention are cruicial to prevent disease progression and reduce the risk of future ischemic or hemorrhagic events. This case emphasizes the necessity of multidisciplinary management and the need for heightened awareness in order to optimize patient prognosis.
